# Quercetin attenuates the reduction of parvalbumin in middle cerebral artery occlusion animal model

**DOI:** 10.1186/s42826-021-00086-0

**Published:** 2021-02-25

**Authors:** Dong-Ju Park, Ju-Bin Kang, Fawad-Ali Shah, Phil-Ok Koh

**Affiliations:** Department of Anatomy, College of Veterinary Medicine, Research Institute of Life Science, Gyeongsang National University, 501 Jinju-daero, 52828 Jinju, South Korea

**Keywords:** Cerebral ischemia, Neuroprotection, Parvalbumin, Quercetin

## Abstract

**Background:**

Calcium is a critical factor involved in modulation of essential cellular functions. Parvalbumin is a calcium buffering protein that regulates intracellular calcium concentrations. It prevents rises in calcium concentrations and inhibits apoptotic processes during ischemic injury. Quercetin exerts potent antioxidant and anti-apoptotic effects during brain ischemia. We investigated whether quercetin can regulate parvalbumin expression in cerebral ischemia and glutamate toxicity-induced neuronal cell death. Adult male rats were treated with vehicle or quercetin (10 mg/kg) 30 min prior to middle cerebral artery occlusion (MCAO) and cerebral cortical tissues were collected 24 h after MCAO. We used various techniques including Western blot, reverse transcription-PCR, and immunohistochemical staining to elucidate the changes of parvalbumin expression.

**Results:**

Quercetin ameliorated MCAO-induced neurological deficits and behavioral changes. Moreover, quercetin prevented MCAO-induced a decrease in parvalbumin expression.

**Conclusions:**

These findings suggest that quercetin exerts a neuroprotective effect through regulation of parvalbumin expression.

## Background

Stroke is the most common cause of disability that has a high mortality rate. It is currently ranked as the third leading cause of death in adults. Cerebral ischemia results from a reduction or blocking of cerebral blood flow, which is mainly caused by thrombosis and embolisms [1]. Moreover, it can cause permanent disability, such as movement disorders and cognitive dysfunction [2]. Cerebral ischemic injury causes pathophysiological changes, including excitotoxicity, depolarization, and inflammation. It consequently leads to neuronal cell damage [3].

Quercetin is a flavonoid that presents in rinds and shoots of plants. In particular, it exists abundantly in various vegetables and fruits, including onions, apple, and apricot [4]. Quercetin offers powerful health benefits because it contains potent antioxidants. It is known that quercetin exerts a protective effect in cardiovascular disease and neurodegenerative diseases [5–9]. Moreover, quercetin performs a neuroprotective function by scavenging reactive oxygen species (ROS) and modulating apoptotic pathways in stroke [10, 11]. Quercetin also protects neurons from oxidative stress and intracellular calcium overload [12].

Maintenance of calcium homeostasis is a critical process for the determining cell fate. A rise in intracellular calcium concentration leads to the impairment of crosstalk signaling between calcium ions and ROS, ultimately causes oxidative damage [13]. Moreover, perturbation of intracellular calcium homeostasis can destroy adherens junctions and tight junctions in blood-brain barrier, which can eventually lead to pathological processes in the brain [14]. Interruption of blood circulation in the brain inhibits the supply of oxygen and glucose to neurons, induces excessive calcium influx, and consecutively leads to neuronal excitation [3]. Parvalbumin is a calcium-buffering protein that plays a vital role in reducing calcium overload in neuronal cells [15, 16]. Parvalbumin contains a paired EF-hand domain that has a high-affinity for calcium binding and it regulates intracellular calcium concentration in neurons [17]. It also plays an important role in presynaptic calcium dynamic signaling and synaptic integration [18]. It is strongly expressed in various brain regions, including cortex, hippocampus, thalamus, midbrain, and cerebellum [19]. In this study, we hypothesized that quercetin modulates parvalbumin expression in ischemic brain injury. Previous studies have been reported the neuroprotective effects of quercetin in stroke, but few studies have been shown that quercetin modulates calcium buffer proteins. Thus, we studied the changes of parvalbumin expression by quercetin treatment in a focal ischemic stroke model.

## Results

**Fig. 1:**
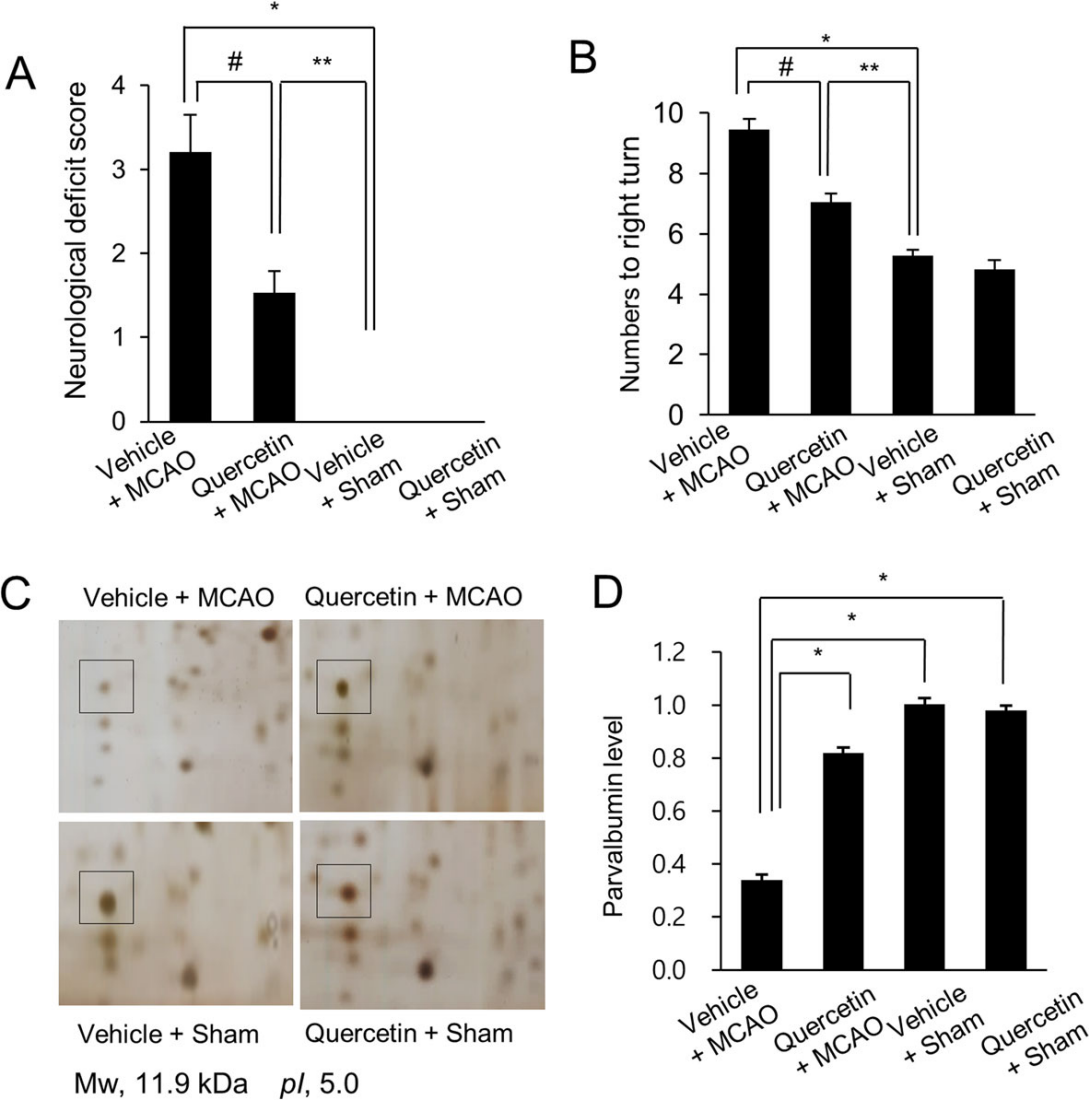
Neurobehavioral scores (**a**), corner test (**b**), and proteomic analysis (**c** and **d**) of parvalbumin in vehicle + middle cerebral artery occlusion (MCAO), quercetin + MCAO, vehicle + sham, and quercetin + sham animals. Quercetin improves the neurological deficit score and tendency of turning towards to the right by ischemic stroke. Data (*n* = 16) are represented as the mean ± S.E.M. * p < 0.01, ** p < 0.05 vs. the vehicle + sham group, *# p* < 0.05 vs. vehicle + MCAO group. Squares indicate parvalbumin protein spots (**c**). Spot intensities were measured by PDQuest software. Spot intensities are reported as a ratio relative to vehicle + sham animals (**d**). Data (*n* = 4) are shown as means ± S.E.M. * p < 0.05. Mw and pI indicate molecular weight and isoelectric point, respectively

**Fig. 2:**
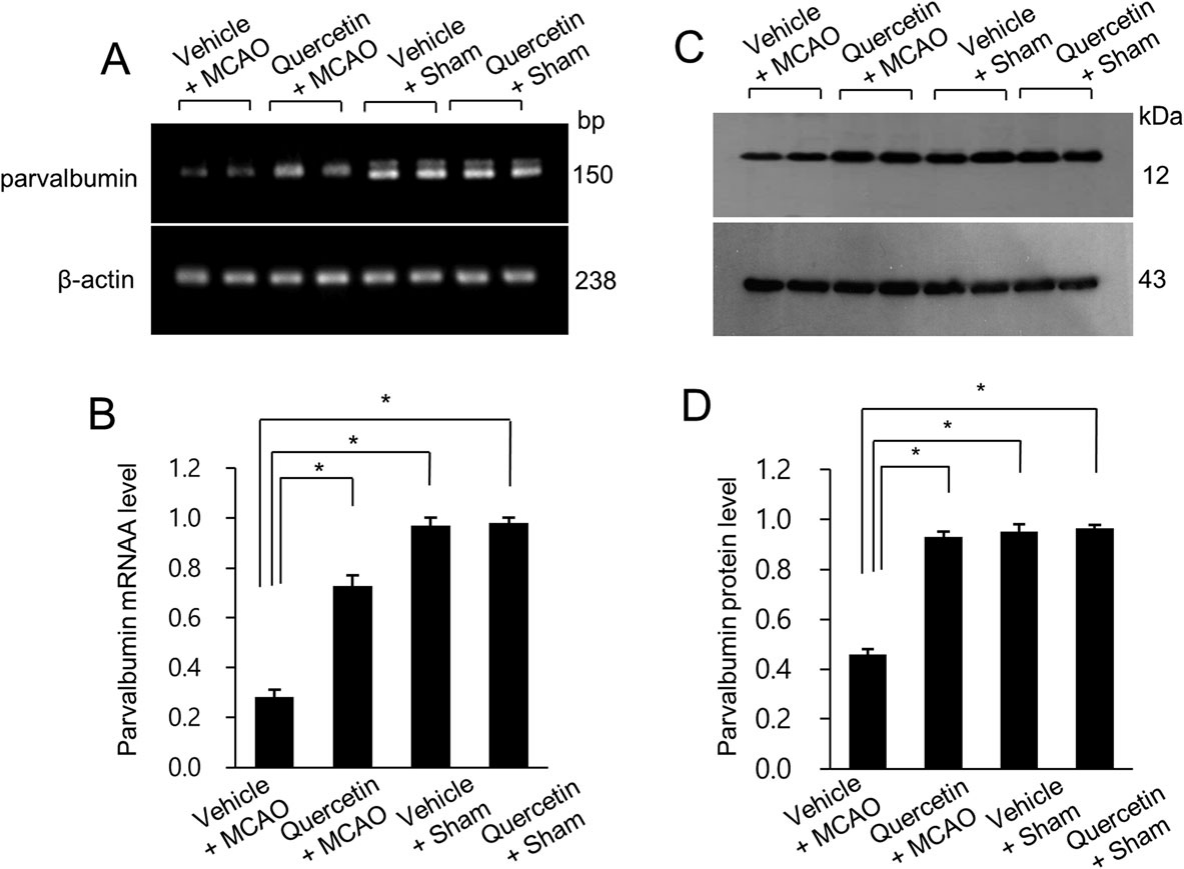
Reverse transcription-PCR (**a** and **b**) and Western blot (**c** and **d**) of parvalbumin in vehicle + middle cerebral artery occlusion (MCAO), quercetin + MCAO, vehicle + sham, and quercetin + sham groups. Densitometric analyses from reverse transcription-PCR and Western blot are represented as a ratio of parvalbumin intensity to β-actin intensity. Each lane represents an individual animal. Data (*n* = 4) are shown as means ± S.E.M. * p < 0.05

**Fig. 3:**
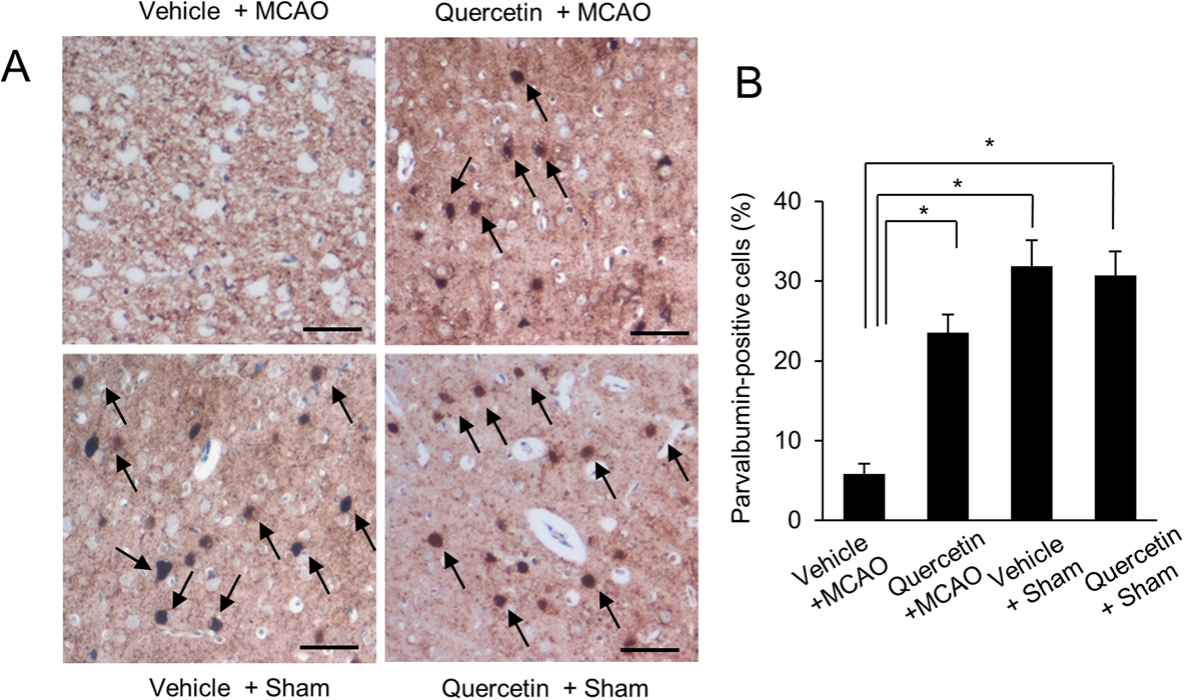
Immuno-staining of parvalbumin in vehicle + middle cerebral artery occlusion (MCAO), quercetin + MCAO, vehicle + sham, and quercetin + sham groups. Arrows indicate positive cells of parvalbumin (**a**). Scale bar = 100 µm. Parvalbumin-positive cells levels were expressed as percentage of the number of parvalbumin positive cells to that of total cells (**b**). Data (*n* = 4) are shown as means ± S.E.M. * p < 0.05

We clearly confirmed the neuroprotective effect of quercetin in MCAO-induced cerebral ischemia using neurobehavioral score evaluation and corner test. MCAO operation induced serious neurological movement deficit in only vehicle-treated animals, whereas quercetin treatment alleviated MCAO-induced movement deficit. Neurological function scores were 3.21 ± 0.44 in vehicle + MCAO animals and 1.53 ± 0.26 in quercetin + MCAO animals, respectively (Fig. 1a). Moreover, the result of corner test showed that quercetin alleviates MCAO injury-induced neurological deficit. The numbers of right turn were 9.44 ± 0.36 and 7.06 ± 0.29 in vehicle + MCAO animals and quercetin + MCAO animals, respectively (Fig. 1b). We screened a decline in parvalbumin expression in the cerebral cortices of vehicle + MCAO animals compared to that of vehicle + sham animals using a proteomic technique. However, this decline was prevented in the presence of quercetin (Fig. 1c). The peptide mass of parvalbumin was 5/103 and the sequence coverage was 40 %. The densitometry levels of the parvalbumin protein spot were 0.34 ± 0.02 and 0.82 ± 0.04 in vehicle + MCAO and quercetin + MCAO animals, respectively (Fig. 1d). RT-PCR and Western blot analyses also clearly showed the changes of parvalbumin expression in the presence of quercetin during ischemic injury (Fig. 2). Parvalbumin transcript levels were decreased in vehicle + MCAO animals compared to that of sham-operated animals. This decrease was prevented in quercetin + MCAO animals (Fig. 2a). Parvalbumin transcripts levels were 0.28 ± 0.03 and 0.73 ± 0.04 in vehicle + MCAO and quercetin + MCAO animals, respectively (Fig. 2b). Quercetin alleviated MCAO-induced decrease in parvalbumin expression (Fig. 2c). Relative parvalbumin protein levels were 0.46 ± 0.02 and 0.93 ± 0.02 in vehicle + MCAO and quercetin + MCAO animals, respectively (Fig. 2d). However, parvalbumin transcript and protein levels were similar between vehicle + sham and quercetin + sham animals. Figure 3 showed that parvalbumin-immunoreactive cells were observed in sham-operated animals regardless of quercetin treatment. However, the number of parvalbumin-positive cells was dramatically decreased in vehicle + MCAO animals, while quercetin treatment attenuated this down-regulation of parvalbumin expression (Fig. 3a). Parvalbumin-positive cells levels were calculated the percentage of the number of positive cells to the number of total cells. Parvalbumin-positive cells levels were 5.88 ± 1.23 and 23.5 ± 2.31 in vehicle + MCAO and quercetin + MCAO animals, respectively (Fig. 3b).

## Discussion

Brain ischemic injury causes oxidative stress and consequently leads to neuronal cell death and neurodegenerative disorders [20, 21]. We previously demonstrated that quercetin exerts a potent neuroprotective effect against MCAO-induced brain injury and glutamate-induced cell death [22]. Quercetin improves MCAO-induced neurological deficit scores and attenuates neuronal damage after MCAO [22, 23]. Quercetin dramatically reduces infarction and protects neuronal cells against cerebral ischemia through modulation of various proteins [22, 23]. Previous reports have showed that quercetin exerts neuroprotective effects in both *in vivo* and *in vitro* experimental models [24, 25]. Also, quercetin protects the blood brain barrier and reduces neuronal damage in brain ischemia by scavenging ROS [26]. We recently reported that quercetin alleviated the decrease of neuronal marker NeuN in the ischemic damaged cerebral cortex [27]. Neuronal markers indicate activation of neurons. Thus, the regulation of NeuN by quercetin represents mitigation of nerve damage in ischemic brain injury. Our previous and current studies explained that quercetin prevented the decrease of neuronal marker expression as well as parvalbumin expression in ischemic brain injury. Our results can demonstrated that quercetin exerts neuroprotective effect by regulating various proteins such as parvalbumin and neuronal marker. This study focused on the regulation of expression of parvalbumin in cerebral ischemic animal models.

Ischemic brain injury causes an increase in intracellular calcium, activates apoptotic signaling pathways, and subsequently leads to nervous system disorders [28–30]. Increased oxidative stress causes intracellular calcium overload, impairs mitochondrial function, and leads to apoptosis [31, 32]. Parvalbumin rescues neurons by attenuating cytotoxic calcium overload in neuronal cell injury [33]. Moreover, over-expression of parvalbumin protects neurons against ischemic injury-induced cell death [34]. Transient forebrain ischemia reduces parvalbumin immunoreactivity in the hippocampus and decline in parvalbumin expression leads to structural damage in neurons [35, 36]. Parvalbumin expression is also reduced in neurodegenerative diseases, such as Alzheimer’s disease and Parkinson’s disease [37, 38]. We identified that quercetin attenuates MCAO-induced a reduction in parvalbumin expression using a proteomic approach. Moreover, Western blot and reverse transcription-PCR analyses showed that quercetin mitigates MCAO-induced a decrease in parvalbumin. In addition, immunohistochemical study elucidated that quercetin treatment alleviates a decrease in the number of parvalbumin-positive cell following MCAO-induced injury. These results demonstrate that quercetin regulates parvalbumin expression in focal cerebral ischemia.

We clearly demonstrated that quercetin can regulate parvalbumin expression. Parvalbumin is a calcium-buffering protein that is abundantly distributed in GABAergic interneurons of the cerebral cortex and hippocampus [39, 40]. Parvalbumin is an essential protein for maintaining calcium homeostasis in neurons by reducing intracellular calcium levels [41]. High levels of intracellular calcium can lead to apoptosis and subsequently cause nervous system disorders [30]. Thus, perturbation of intracellular calcium homeostasis is considered to be a major pathological mechanism in stroke [42, 43].

## Conclusions

This study clearly showed that quercetin has a neuroprotective effect on deteriorated neurological function in MCAO injury. Furthermore, we demonstrated that quercetin alleviates down-regulation of parvalbumin expression during ischemic brain injury. Therefore, our findings suggest that quercetin might exert its neuroprotective functions by modulation of parvalbumin expression in MCAO-injured animals.

## Methods

### Experimental animals

Adult male Sprague-Dawley rats (220–230 g, *n* = 64, Samtako Co, Osan, Korea) were housed at controlled light condition (12 h light/12 h dark cycle, light on at 6:00 AM to 6:00 PM) and temperature (25°C). Feed and water were freely supplied. All experimental processes were taken to minimize the suffering of animals, approved by the Institutional Animal Care and Use Committee of Gyeongsang National University, and carried out provided guideline. Animals were randomly divided into four groups as follows: vehicle + middle cerebral artery occlusion (MCAO), quercetin + MCAO, vehicle + sham, and quercetin + sham. Quercetin (Sigma-Aldrich, St. Louis, MO, USA) was dissolved in 0.1 % dimethyl sulfoxide (DMSO) with phosphate buffered saline (PBS). Quercetin (10 mg/kg) or vehicle was intraperitoneally injected 30 min before MCAO operation [11]. Vehicle animals were administered only a solvent solution without quercetin.

### Middle cerebral artery occlusion surgical procedure

MCAO was performed through insertion of intraluminal nylon to induce focal cerebral ischemia [44]. Animal was anesthetized with Zoletil (Virbac, Carros, France) at 50 mg/kg and placed on surgical plate with supine position. A warming pad was used to maintain body temperature during surgical process and midline of neck skin region was incised. Right common carotid artery (CCA), external carotid artery (ECA), and internal carotid artery (ICA) were exposed by separation of surrounding tissues. CCA was temporally blocked with a microvascular clip and ECA was tied. A 4/0 nylon filament with rounded tip by flame heating was inserted into the incision of ECA. Inserted filament was advanced to the lumen of the ICA until a slight resistance was felt. The origin of middle cerebral artery was occluded by the end of nylon filament. After the occlusion, nylon filament was ligated with ECA to remain stationary and skin incision was sutured with silk. Sham animals as control were operated by same procedure except the nylon insertion step. Neurofunction and corner tests were performed 24 hours after MCAO surgery and started at 10:00 AM. Animals were sacrificed after behavior test and brain tissues were collected. Whole brains were fixed in 4 % neutral buffered paraformaldehyde for morphological study and cerebral cortices were isolated from brain tissues and kept at -70°C for protein and RNA works.

### Neurological functional test and corner test

Neurological functional test was carried out as described methods [45, 46]. Neurological function was scored by an investigator who was blind to the experimental design. Criteria of neurological functional test was as follows: 0 indicates normal neurological function status; 1 indicates mild neurological deficit status (forelimb of the contralateral side is flexed when the animal is lifted by tail); 2 indicates moderate neurological deficit status (circling to the contralateral side when moving forward); 3 indicates severe neurological deficit status (falling to the contralateral side when moving forward); 4 indicates unconscious state and no movement. In addition, corner test was performed to evaluate sensorimotor dysfunction [47]. Animals were placed between two boards with dimensions of 30 × 20 × 1 cm^3^. Two boards were facing each other at an angle of 30° with a slight gap to guide the animal towards the edge. Animals were placed facing the corners and moved forward, and both vibrissae were simultaneously stimulated. After stimulation, animals turned to the left or right direction and came out in the opposite direction of the corners. Animals were trained for corner test seven days prior to MCAO surgery. The number of turns to the right (ipsilateral side of the damaged brain) from the corner in ten times trial was measured and incomplete turn was excluded from the measurement.

### Two Dimensional Electrophoresis (2DE)

Right cerebral cortexes (100 mg) were homogenized with 200 µl of lysis buffer (8 M urea, 4 % CHAPS, ampholytes, and 40 mM Tris-HCl) and lysed by sonication for 3 min. Lysate was centrifuged at 16,000 g for 20 min at 4°C and supernatant was collected. And then supernatant was mixed with same volume of 20 % trichloroacetic acid and centrifuged 16,000 g for 15 min at 4°C. After centrifugation, supernatant was discarded and protein pellet was washed with 90 % acetone. Dried protein pellet was dissolved with lysis buffer and protein concentration was measured by Bradford protein assay kit (Bio-Rad, Hercules, CA, USA). For first dimensional isoelectric focusing (IEF), protein sample (50 µg) was mixed with rehydration buffer [8 M urea, 2 % CHAPS, 20 mM dithiothreitol (DTT), 0.5 % immobilized pH gradient (IPG) buffer, and bromophenol blue], loaded on IPG gel strip (17 cm, pH 4–7, Bio-Rad), and rehydrated for 13 h. IPG strip containing protein sample was run at 250 V for 15 min, 10,000 V for 3 h, and then 10,000 V to 50,000 V on Ettan IPGphor 3 System (GE Healthcare, Uppsala, Sweden). Strips were immersed with equilibration buffer [6 M urea, 30 % glycerol, 2 % sodium dodecyl sulfate, and 50 mM Tris-HCl (pH 8.8)] containing 1 % DTT for 15 min and subsequently incubated with same solution that containing 2.5 % iodoacetamide instead of DTT. For second dimensional electrophoresis, strip was overlaid onto 12 % acrylamide gel and run at 15 mA for 15 h using Protean-II XI electrophoresis equipment (Bio-Rad). Acrylamide gels were fixed in solution containing 12 % acetic acid and 50 % methanol, and washed in 50 % ethanol. They were sensitized with 0.02 % sodium thiosulfate, stained in 0.2 % silver nitrate solution, and developed with 2 % sodium carbonate. Silver-stained gel image was digitized by Agfa ARCUS 1200™ (Agfa-Gevaert, Mortsel, Belgium) and analyzed by PDQuest 2-D analysis software (Bio-Rad). Targeted protein spots were removed from gel, destained, and freeze-dried. They were alkylated and digested with trypsin-containing buffer. Digested peptide samples were mixed with matrix solution containing 0.02 % α-cyano-4-hydroxycinnamic acid and placed on metal plate. Voyager System DE-STR MALDI-TOF mass spectrometer was used to analyze peptide samples and analyzed peptide was detected by MS-Fit and ProFound programs. Based on peptide mass data, peptide sequences were obtained from SWISS-prot and NCBI databases.

### Reverse Transcription‐Polymerase Chain Reaction (RT-PCR)

Total RNA was extracted from right cerebral cortex using Trizol Reagent (Life Technologies, Rockville, MD, USA) and reverse transcribed into complementary DNA using Superscript III firststrand system (Invitrogen, Carlsbad, CA, USA) according to the recommended protocol of manufacturer. Parvalbumin-coded gene was detected from synthetized complementary DNA and amplified by PCR method. Used primer sequences were as follows: parvalbumin, forward primer: 5-AAGAGTGCGGATGATGTGAAG-3’, reverse primer: 5-AGCCATCAGCGTCTTTGTTT-3’; β-actin, forward primer: 5-GGGTCAGAAGGACTCCTACG-3’, reverse primer: 5-GGTCTCAAACATGATCTGGG-3’. PCR was performed for thirty cycles and PCR conditions were as follows: initial denaturation and polymerase activation at 94℃ for 5 min, denaturation at 95°C for 30 sec, primer annealing at 52.5°C for 30 sec, and elongation at 72°C for 30 sec. Amplified target DNA products were mixed with LoadingSTAR dye (Dyne Bio, Seongnam, Korea) and loaded on 1 % agarose gel. Loaded product bands were visualized by ultra-violet light exposure.

### Western blot analysis

Immunoblot analysis was carried out as a previously described method [48]. Samples from right cerebral cortexes were homogenized in lysis buffer [1 % Triton X-100 and 1 mM EDTA in PBS (pH 7.4)] using a tissue homogenizer and centrifuged at 16,000 g for 20 min at 4°C. Supernatant was isolated and protein concentration of each sample was calculated with bicinchroninic acid (BCA) protein assay kit (Thermo Fisher Scientific, Waltham, MA, USA) according to the manufacturer’s manual. Protein (30 µg) from each sample was loaded on 10 % sodium dodecyl sulfate poly-acrylamide gel electrophoresis and electrophoresed until dye went down to the bottom. Protein was transferred to a poly-vinylidene fluoride membrane and membranes were incubated with 5 % skim milk in tris-buffer saline containing 0.1 % Tween-20 (TBST) for 1 h. Membranes were washed three times with TBST for 10 min and reacted with following primary antibodies: anti-parvalbumin (1:1,000; Thermo Fisher Scientific) and anti-β-actin (1:1,000; Santa Cruz Biotechnology, Santa Cruz, CA, USA) overnight at 4°C. They were washed with TBST and incubated with horseradish peroxidase-conjugated anti-rabbit IgG or anti-mouse IgG (1:5,000; Thermo Fisher Scientific) for 2 h. Membranes were washed with TBST and reacted with enhanced chemiluminescence Western detection reagents (GE Healthcare) according to the manufacturer’s manual.

### Immunohistochemistry

Fixed brain tissues were washed with tap water, dehydrated with graded ethyl alcohol (70–100 %), and cleaned with xylene. Brain tissues were embedded with paraffin and sectioned with 4 µm thickness. Sections were deparaffinized with xylene, rehydrated with graded ethyl alcohol (100–70 %), and immersed in sodium citrate buffer (0.01 M, pH 6.0) using a microwave oven for antigen retrieval. Sections were treated with 1 % hydrogen peroxide in methanol for 10 min to exhaust peroxidase activity. They were incubated with normal goat serum for 1 h to block non-specific reaction and reacted with anti-parvalbumin primary antibody (1:100, Thermo Fisher Scientific) overnight at 4°C. They were washed with PBS and treated with biotin-conjugated secondary antibody for 2 h. After washing with PBS, sections were incubated with avidin-biotin-peroxidase complex (Vector Laboratories Inc, Burlingame, CA, USA) for 2 h. Brain sections were stained with 3, 3’-diaminiobenzidine (DAB, Sigma-Aldrich) for 15 min and hydrogen peroxide was added. Sections were counterstained with hematoxylin, washed with water, dehydrated with graded ethyl alcohol (70–100 %), and cleaned with xylene. Stained tissues were mounted with permount solution (Thermo Fisher Scientific) and observed with Zeiss Axioskop light microscope (Carl Zeiss, Jena, Germany). Five fields from cerebral cortex region were randomly selected and the number of parvalbumin-positive cells was counted. Parvalbumin-positive cells levels were expressed as percentage of the number of parvalbumin positive cells to that of total cells.

### Statistical analysis

Data were presented as means ± standard error of mean (S.E.M.). Results of each groups were compared by two-way analysis of variance (ANOVA) followed by *post-hoc* Scheffe’s test. *p* < 0.05 was considered to be statistically significant.

## Data Availability

The data that support the findings of this study are available on request from the corresponding author on reasonable request.

## Abbreviations

2DE: Two dimensional electrophoresis
BCA: Bicinchroninic acid
CCA: Common carotid artery
DAB: 3,3′-diaminobenzidine
DMSO: Dimethyl sulfoxide
DTT: Dithiothreitol
ECA: External carotid artery
ICA: Internal carotid artery
IEF: Isoelectric focusing
IPG: Immobilized pH gradient
MCAO: Middle cerebral artery occlusion
PBS: Phosphate-buffered saline
ROS: Reactive oxygen species
RT-PCR: Reverse-transcript polymerase chain reaction
PVDF: Poly-vinylidene fluoride
TBST: Tris-buffered saline containing 0.1 % Tween-20
